# Knowledge, attitudes and perception of communities on mosquitoes and its control practices in Lagos State, Nigeria

**DOI:** 10.11604/pamj.2021.38.44.22572

**Published:** 2021-01-15

**Authors:** Ifeoluwa Kayode Fagbohun, Emmanuel Taiwo Idowu, Ayooluwa Olumide Onafuwa, Adeniyi Kazeem Adeneye, Adedapo Olufemi Adeogun, Otubanjo Olubunmi Adetoro

**Affiliations:** 1 Department of Zoology, University of Lagos, Akoka, Yaba, Lagos, Nigeria,; 2 Public Health and Epidemiology Department, Nigeria Institute of Medical Research, Yaba, Lagos, Nigeria

**Keywords:** Mosquitoes, knowledge, perception, insecticides, control

## Abstract

**Introduction:**

evidence-based mosquito control strategy is important for efficient and effective delivery of mosquito control interventions. This is hinged on effective community participation and thorough understanding of the Knowledge Attitude and Practices (KAPs) to achieve desired result. Such community dynamics are often understudied. We designed this study to assess the perception of four local communities on aspects of mosquito behavior, prevention and control in Lagos State, Nigeria.

**Methods:**

a cross-sectional survey was carried out using pretested semi-structured questionnaires to assess socio-demographic factors and KAPs in Kosofe, Alimosho, Ibeju-Lekki and Badagry Local Government Areas of Lagos State, Nigeria. Data analysis was carried out using IBM SPSS version 23.

**Results:**

a total of 746 questionnaires were analyzed. Socio-demographic profile of the sampled population reveals that majority of the study population (73.1%) was between 18 and 40 years which constitute 49% males and 51% females. The knowledge of mosquito as a disease vector was high among the respondents which correlates with their level of education (P<0.05). The use of insecticide aerosols and Insecticides Treated Nets (ITNs) are the main control measures employed for mosquito control by respondents. Cost, convenience of usage and awareness majorly influenced the type of control measures that respondents adopt. Reasons such as not being easy to setup, skin irritation and the filling of being caged are reasons why some individuals do not use ITNs. Indoors, 32.4% of the respondents indicate the use of dichlorvos (DDVP) for household control of mosquitoes.

**Conclusion:**

the knowledge of mosquito control is high among middle aged individuals in Lagos State. Insecticide aerosols and ITNs are two major mosquito control methods used with DDVP insecticides frequently used indoors. This can inform the design of appropriate control methods in Lagos State.

## Introduction

Mosquito-borne diseases pose serious threat to humans and communities in different parts of the world. They transmit human parasitic and viral infections including malaria, dengue, lymphatic filariasis, chikungunya, Zika virus disease, yellow fever, Japanese encephalitis and West Nile fever. Morbidity and mortality rates of these diseases are often disproportionately high in poorer populations and in some cases, people who survive these diseases can be left permanently disabled or disfigured [1]. However, the risk of infection for certain parasitic and viral pathogens is particularly high in towns and cities where Aedes and Culex mosquitoes proliferate because of favorable habitats. Most control efforts are tailored towards Indoor Residual Spray (IRS) and Long-Lasting Insecticide Treated Nets (LLINs) use. Moreover, the success of these two major methods largely depend on acceptance and community participation which varies from one community to another and hinge on the understanding of community believes and dynamics. In Nigeria, evidence-based researches are often emphasized, while most efforts are geared towards LLINs and IRS interventions, it is important to pay more attention to community dynamics before and after these interventions for effective community understanding.

To achieve effective and efficient mosquito control it is of utmost importance to understand community dynamics. These community dynamics in relation to vector control largely depends on people´s knowledge and attitude and practices towards mosquito borne diseases. This is crucial for the development integrated approach for community vector control. Most organized vector control strategies require public support of one kind or another and the extent of people´s cooperation can determine the success or failure of the entire campaign [2]. Obtaining community support and participation for disease control measures involves the understanding of the knowledge, attitude and practices of affected individuals [3]. There is a paucity of information on the people´s knowledge of the different mosquito species, their public importance and control measures being applied in communities within Lagos State, Nigeria. This study was therefore designed to understand knowledge, attitude and perception of respondents in Lagos State to mosquitoes and mosquito-borne diseases.

## Methods

**Study area:** the study was carried out in four (4) Local Government Areas (LGAs) of Lagos State, Nigeria including: Eti-Osa LGA (6°26´34; N, 3°28´29 E), situated within the southern area of Lagos State, just below the Lagos lagoon. Kosofe LGA situated at 6°45'N, 3°4'E and 35 meters´ above sea level. Alimosho LGA (6°36'38"N 3°17'45"E), the largest local government in Lagos with 1,288,714 inhabitants according to the National Population Census (NPC) of 2006 and Badagry LGA (6°25'N 2°53'E); a coastal town located between Metropolitan Lagos, and the border with Republic of Benin at Seme.

**Study design and data collection:** a cross-sectional survey was carried out between January and December 2018 using pretested semi-structured questionnaires that probed into the socio-demographic factors, knowledge attitude and perception regarding mosquito diversity, mosquito-borne diseases and control measures. Questionnaires were administered on randomly selected individuals from two coastal and less densely populated LGAs (Badagry and Ibeju-Lekki) and two main lands and densely populated LGAs (Kosofe and Alimosho) The questionnaire was administered to the head of the household or an available responsible adult was interviewed. Only one person per household was interviewed. All participants gave verbal consent.

**Sample size determination:** for estimating a population proportion with specified relation precision, the formula below was used.

n=Z21−α/21−PPε2

Where n = sample size, 1-α = confidence level, Z_1-α/2_ = represents the number of standard errors from the mean, a function of confidence level, P = anticipated population proportion, e = relating precision [4]. Since P value from previous studies on this present study is not available an anticipated P value of prevalent knowledge regarding mosquito is taken as (50%). At p = 0.50 (50%) and e = 10%, a sample size of 625 would be needed [4]. To improve the precision further, the sample size was taken 750 household respondents. The total sample divided into four parts of about 150 each to have a fair representation from different LGAs.

**Data analysis:** data collected were carefully entered and checked in Microsoft Excel and descriptive statistics were computed using and IBM SPSS Statistics 23.

## Results

The socio-demographic characteristics of the respondents are presented in Table 1. Most (73.1) respondents were aged 18-40 years. Over half (53%) of the respondents had tertiary education while only 6.7% had no formal education. Majority of the surveyed population were gainfully employed (38.2%) and students (34%), and the income profile showed that 33% of the population earned above the minimum wage.

**Table 1:** socio-demographic characteristics of respondents in Lagos State

Variables		Frequency (N)	Percentage (%)
**Location**	Kosofe	201	26.9
	Alimosho	193	25.9
	Badagry	146	19.6
	Ibeju-Lekki	206	27.6
**Age group**	Below 18	66	8.8
	18-40	545	73.1
	Above 40	115	15.4
**Sex**	Male	364	48.8
	Female	377	50.5
**Educational status**	No formal education	50	6.7
	Primary	30	4.0
	Secondary	216	29.0
	Tertiary	424	56.8
**Marital status**	Single	506	67.8
	Married	198	26.5
	Divorced	10	1.3
	Separated	15	2.0
**Employment status**	Student	254	34.0
	Employed	285	38.2
	Self employed	131	17.6
	Unemployed	56	7.5
**Income per month**	No income	29	3.9
	Below 18,000	118	15.8
	18,000 and above	246	33.0

**Figure 1:**
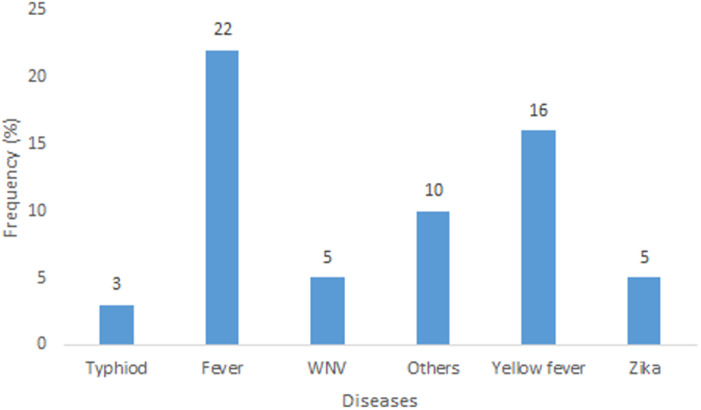
respondents perception of other diseases transmitted by mosquito

**Figure 2:**
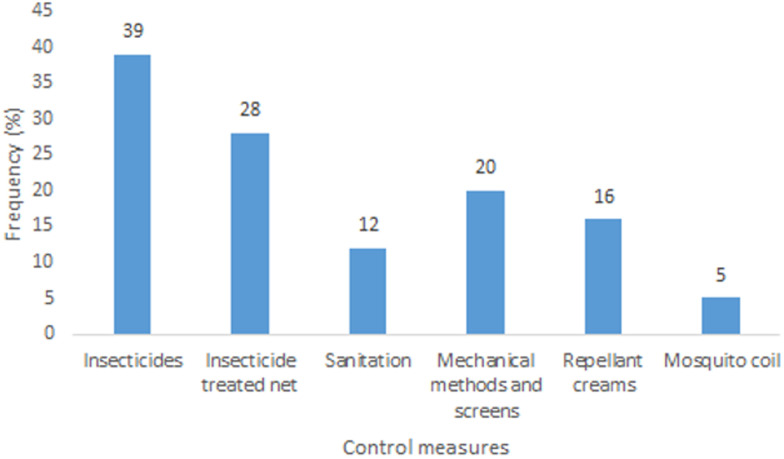
preferred personal protection measures against mosquitoes employed by respondents in Lagos State

**Figure 3:**
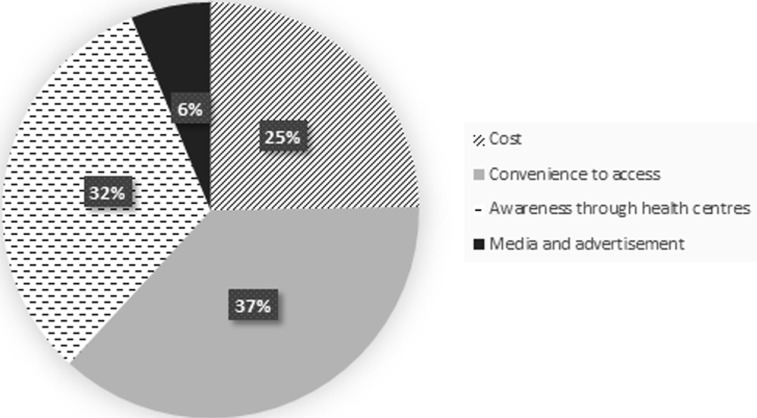
reason for choice of control among surveyed populations in Lagos State

**Figure 4:**
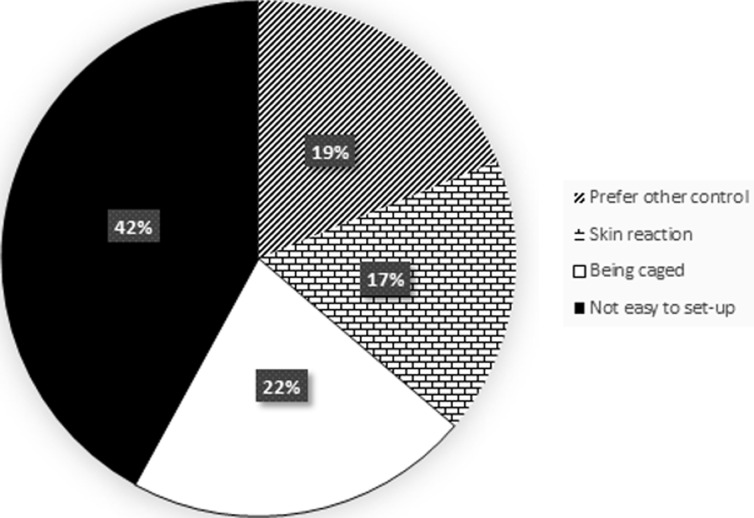
reason for not using LLINs among survey populations in Lagos State

Knowledge of mosquito as a nuisance biter, vector of malaria and several other infectious diseases was high among the respondents. The knowledge of mosquitoes among the surveyed individuals showed statistical significance in relation to their level of education (P < 0.05) (Table 2). Respondents mentioned fever and yellow fever as the main other infectious diseases transmitted by mosquitoes apart from malaria, other diseases perceived to be transmitted by mosquitoes include: Zika, typhoid fever, and West Nile virus (Figure 1). The use of insecticides (in form of aerosols and topical insecticides) and Insecticides Treated Nets (ITNs) are the main control measures being applied by individuals. Others include repellants creams, sanitation, mechanical methods and mosquito coils (Figure 2). Cost (25%), the convenience of use (37%) and awareness through health workers and centers (32%) significantly influenced the type of control measures that individuals adopted (Figure 3). Perception of not being easy to setup (42%), the feeling of being caged (22%) and skin irritation (17%) are reasons why some individuals don´t use LLINs while the rest (19%) prefer the use of mosquito control measures (Figure 4). About 32% of the people who used insecticides at home also indicated the use of DDVP for the control of control mosquito.

**Table 2:** knowledge of mosquito in relation to educational status among respondents in Lagos State

Variables	Educational status	Frequency (%)	
**Knowledge of mosquitoes**	No formal education	45(91.8)	0.000
	Primary	26 (86.7)	
	Secondary	86.1 (86.1)	
	Tertiary	414(99)	
	Others	1(7.1)	
**Cause harm to humans**	No formal education	38(80.9)	0.000
	Primary	10(40)	
	Secondary	183(91)	
	Tertiary	416(99.8)	
	Others	9(100)	
**Transmission of malaria**	No formal education	34(91.9)	0.305
	Primary	10(90.0)	
	Secondary	163(93.1)	
	Tertiary	395(96.3)	
	Others	14(100)	
**Transmission of other diseases**	No formal education	7(20.6)	0.323
	Primary	0(0)	
	Secondary	46(32.9)	
	Tertiary	109(28.3)	
	Total	172(29.6)	

The usage of insecticides and Long Lasting Insecticides Treated Nets among the surveyed population was 50.8% and 66.1% respectively. Majority (82.4% and 76.7%) are of the opinion that insecticides and LLINs are effective in the control of mosquitoes. Most (45.5%) of the individuals that use LLINs for mosquito control purchased the nets while 30.8% of them got it through free distribution. The distribution of free LLINs is the main control effort that has been done in the surveyed Local Government Areas (Table 3).

**Table 3:** usage of insecticides, LLINs and control programmes conducted in respondents location in Lagos State

Variables		Frequency (N)	Percentage (%)
**Usage of insecticides**		220	50.8
**Effectiveness of insecticides**		615	82.4
**Usage of LLINs**		493	66.1
**How LLINs was acquired**	**Purchased**	339	45.4
	**Free**	230	30.8
**Effectiveness of LLINs**		572	76.7
**Perception if mosquito can be controlled**		538	72.1
**Control programme in area**		298	39.9
**Programme type**	**Larviciding**	31	4.2
	**Free LLINs**	273	36.6
	**IRS**	37	5
**Effectiveness of control programme**		220	69.4

## Discussion

To develop a successful and effective control strategy, it is crucial to gain community participation and an understanding of current knowledge and practices regarding mosquito control in the targeted communities [3, 5]. Most mosquito KAP related studies are focused on mosquito-borne diseases such as malaria and dengue, only a few studies have assessed people´s perception and attitude towards mosquito and their control.

Results from this present study reveal that the knowledge of mosquito among respondents as an insect and a vector of malaria and other infectious diseases is high among the surveyed population. Other than malaria, respondents believed that mosquitoes transmit yellow fever, Zika, West Nile virus and typhoid, similar to a previous report from Jamnagar district India [2]. The use of insecticides in homes, insecticide-treated nets, sanitation including clearing of stagnant water, repellant creams, and mosquito coils are some of the preferred personal protection measures individuals use to prevent mosquito bite. A large proportion (32.4%) of the respondents indicated they use DDVP, an organophosphate insecticide not approved by WHO for use in public health and that has been found harmful to human health for the control of mosquito [6]. The extensive and indiscriminate use of insecticides like DDVP at homes in the control of mosquitoes could lead selection pressure thereby, affecting the efficacy and effectiveness of IRS as a result of insecticides resistance. In this study, most people that do not use LLINs gave reasons such as, difficulty in setting it up, having the feeling of being caged, skin reactions and preference of other control measures. Many (66.1%) of the survey individuals indicated the use of LLINs slightly higher than about 60% report by World Health Organization in 2017 [7]. Similarly, the Nigerian Demographic and Health Survey of 2018 recorded a total ITN coverage per household of 60.2%, though the coverage in Lagos State was 29.3% [8]. According to the data from this study, the media, health centers and cost play major role in the type of control measures applied to mosquitoes, similar to previous studies in different parts of Nigeria [9, 10] and Ghana [11].

## Conclusion

The knowledge of mosquitoes as a nuisance biter and vector of different diseases was high among the surveyed population. There is a need to further sensitize the people on the importance of environmental sanitation, diseases transmitted by mosquitoes, the use of appropriate insecticides for mosquito control and the benefit of proper usage of LLINs in the control of mosquito-borne diseases.

### What is known about this topic

- Mosquitoes are known vectors of different infectious diseases in Nigeria including malaria, yellow fever, lymphatic filariasis etc;
- The use of insecticides and ITNs is a common means of control mosquitoes in Nigeria;
- Resistance has been reported to different classes of insecticides in mosquitoes from Nigeria.

### What this study adds

- This study provides information of the widespread of DDVP an unapproved insecticide for mosquito control in Lagos State, Nigeria;
- Provides the information on the usage of LLINs and the challenges in the utilization of this mosquito control measure among respondents in Lagos State;
- The study also provides information on the knowledge of Lagos State residents on mosquitoes as a vector of different disease and attitude towards the different control measures.

## References

[1] WHO (2017). Global vector control response 2017-2030.

[2] Dhaduk KM, Dipesh P, Yadav SB (2013). A community level KAP study on mosquito control in Jamnagar district. Natl J Community Med.

[3] Otubanjo OA, Idowu ET, Fagbohun IK, Okorie NE, Oke BI (2016). Tungiasis in rural communities of Badagry Local Government Area, Lagos State, Nigeria. Zool.

[4] Lwanga SK, Lemeshow S (1991). Sample size determination in health studies.

[5] Kumaran E, Doum D, Keo V, Sokha L, Sam B, Chan V (2018). Dengue knowledge, attitudes and practices and their impact on community-based vector control in rural Cambodia. PLoS Negl Trop Dis.

[6] Eze JN, Ndu IK, Edelu BO (2018). Teenage organophosphate insecticide poisoning?: An ugly trend in Enugu Nigeria. J Community Med Prim Heal Care.

[7] WHO (2017). World Malaria Report 2017.

[8] NPC ICF (2019). Nigeria Demographic and Health Survey Key Indicators Report (Abuja Nigeria, and Rockville, Maryland, USA). National Population Commission and The DHS Program.

[9] Ezeigbo OR, Ejike EN, Nwachukwu I (2016). Insecticide-treated Bed Net (ITN): Ownership and Usage in the Control of Malaria in Abia State, Nigeria. Am J Epidemiol Infect Dis.

[10] Komomo EA, Egena R, Irene C, Akintoye OA, Peter OA (2016). Assessment of the Utilization of Insecticide Treated nets (ITNs) in Metropolis, Calabar State, Cross River. J Heal Med Nurs.

[11] Nyavor KD, Kweku M, Agbemafle I, Takramah W, Norman I, Tarkang E (2017). Assessing the ownership, usage and knowledge of Insecticide Treated Nets (ITNs) in Malaria Prevention in the Hohoe Municipality, Ghana. Pan Afr Med J.

